# A Genetic Algorithm and Fuzzy Logic Approach for Video Shot Boundary Detection

**DOI:** 10.1155/2016/8469428

**Published:** 2016-03-27

**Authors:** Dalton Meitei Thounaojam, Thongam Khelchandra, Kh. Manglem Singh, Sudipta Roy

**Affiliations:** ^1^CSE Department, National Institute of Technology Silchar, Assam, India; ^2^CSE Department, Assam University Silchar, Assam, India; ^3^CSE Department, National Institute of Technology Manipur, Manipur, India

## Abstract

This paper proposed a shot boundary detection approach using Genetic Algorithm and Fuzzy Logic. In this, the membership functions of the fuzzy system are calculated using Genetic Algorithm by taking preobserved actual values for shot boundaries. The classification of the types of shot transitions is done by the fuzzy system. Experimental results show that the accuracy of the shot boundary detection increases with the increase in iterations or generations of the GA optimization process. The proposed system is compared to latest techniques and yields better result in terms of *F*1score parameter.

## 1. Introduction

With the growth of the Internet, the generation of multimedia contents is also increasing. This leads to the problem of effective utilizing and managing the video data. Effective utilizing and managing of the multimedia contents need effective indexing and retrieval system. This is much more difficult in the case of video. For an effective video retrieval system, the content of the video should be understood so that proper indexing system can be created for better video retrieval. The content of the video can be taken by first performing the video segmentation, dividing the video into meaningful shots, and analyzing each feature of the segments (shots) which is the key feature of each segment. A scene is a combination of more than one shot with different camera angles or a combination of similar shots.

In video segmentation (shot boundary detection), the video is divided into meaningful scenes so that each scene can be analyzed for finding the key feature(s). Shot boundary detection mainly consists of finding the two types of transitions abrupt transition and gradual transition [1, 2]. Abrupt transition (also known as hard cut) is the sudden change of the consecutive frames in a video which marks the scene change due to sudden release of the camera rolling. Gradual transition (also known as soft cut) is of four types: fade-in, fade-out, dissolve, and wipe transitions. All these gradual transitions are a result of the editing effect in a video. Fade-in and fade-out are caused by the lightness value. In fade-in, a picture appears slowly from a darker (usually black) empty frame. In fade-out, a picture slowly diminishes to an empty frame (usually black frame). Dissolve and wipe transition is an effect due to overlapping of the current scene and the future scene. In dissolve, the overlapping is done in such a way that the current scene starts disappearing and the future scene starts appearing simultaneously. In wipe, the overlapping is done in such a way that the future scene grows over the current scene until the future scene appears completely.

## 2. Related Works 

Many researchers [1–3] have tried to detect the transitions (known as shot boundary detection or temporal video segmentation) in a video in compressed and uncompressed domain. MPEG (Motion Picture Expert Group) provides video formats which provide a large area of analyzing frame features in the compressed domain using motion vectors [4], Discrete Cosine Transform coefficients [5], and so forth. The frame feature extraction can be globally and locally. Global feature extraction considers the whole feature of the frame such as the pixel value [6]. Local feature extraction considers some regions of the frame and the features in that region are only taken or in other senses the necessary/important features of the whole frame are considered. MSER [7], SURF [8], and so forth are some of the popular local feature descriptor used for shot boundary detection. These features are extracted from each frame of the video and calculate the differences between consecutive frames to find out the transitions. The gradual transitions are rather difficult than the abrupt transition as it may have the same effect with large object motion and camera motion [1]. Thus, it is necessary to extract features which give less/no effect with large object motion, camera motion, or lighting effect.

Intensity histogram and Color Histogram Difference are of the effective, simple, and widely used methods for shot boundary detection in the uncompressed domain which is not sensitive to motion [6]. In [10, 11], SVD is applied to frame histogram matrix and a similarity measure is applied to find out the abrupt and gradual transitions. In [10], *n* consecutive frames between two frames are skipped for analysis, which reduces the computational time drastically. In [9], HSV color histogram and an adaptive threshold are used for shot boundary detection and also the algorithm can detect flashes. In [8], entropy and SURF features are used to find the cut and gradual transitions where the intensity histogram is used to calculate the entropy of a frame.

Genetic Algorithm [12, 13] and Fuzzy Logic [6, 14, 15] have been used for shot boundary detection. In [16], color histogram is generated using Fuzzy Logic for abrupt and gradual transition detection. In [17], an Adaptive Fuzzy Clustering/Segmentation (AFCS) algorithm is proposed and the fuzzy clustering algorithm is used for image segmentation where it takes into account the inherent image properties like the nonstationarity and the high interpixel correlation. A Multiresolution Spatially Constrained Adaptive Fuzzy Membership Function is used for tuning the AFCS. In [18], Genetic Algorithm is used to generate the membership function of the fuzzy system for image segmentation.

In this paper, we introduced a method of shot boundary detection using Fuzzy Logic system optimized by GA. Fuzzy system is used to classify the video frames into different types of transitions (cut and gradual) using normalized Color Histogram Difference. GA is used as optimizer to find the optimal range of values of the fuzzy membership functions. The result shows that the combination of this feature is efficient and the accuracy increases with increase in iterations/generations of GA.

The paper is organized as follows.  Section 3 explains the feature extraction of the system. A detail explanation of the GA optimized fuzzy system to find out that the range of values of the membership functions is given in Section 4. Experimental Results and Discussion and Conclusion are given in Sections 5 and 6, respectively.

## 3. Feature Extraction

This section discussed the feature extraction used in our proposed system.

### 3.1. Color Histogram Difference

Color histogram is a global feature extraction technique which is one of the simplest and widely used image feature extractions for shot boundary detection [19]. It is nonsensitive to motion [6, 14]. In [6], the normalized color histogram between two frames, say *i*th and (*i* + 1)th frames, in a video is defined as follows:
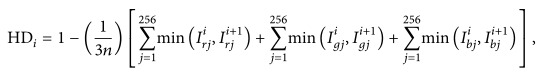
(1)where *n* is the number of pixels in a frame, *I*_*rj*_^*i*^ is the number of red pixels of *i*th frame in *j*th bin, and vice versa. *r*, *g*, and *b* represent red, green, and blue components of a frame. It is observed that (1) yields a value with an interval [0, 1]. HD_*i*_ yields a value 0 when the *i*th and (*i* + 1)th frames are same and the HD_*i*_ value goes on increasing as the similarity between *i*th and (*i* + 1)th frames decreases.

## 4. Fuzzy Logic System with GA Optimization for Finding the Value Range of the Membership Function

Genetic Algorithm (GA) is used as optimizer to find optimal values of the membership functions of the Fuzzy Logic system [20, 21]. The steps are shown as follows.

### 4.1. Fuzzification

First we define the input and output variables of the fuzzy system.

The input variables are

(a) HD_*i*_ is with linguistic values negligible (N), small (S), significant (Sig), large (L), and huge (H);
 Variable HD_*i*_ is the histogram difference value which is the difference between *i*th and (*i* + 1)th frames and is computed using normalized histogram intersection;
(b) HD_*i*−1_ is with linguistic values negligible (N), small (S), significant (Sig), large (L), and huge (H);
 Variable HD_*i*−1_ is the histogram difference value which is the difference between (*i* − 1)th and *i*th frames;
(c) HD_*i*+1_ is with linguistic values negligible (N), small (S), significant (Sig), large (L), and huge (H);
 Variable HD_*i*+1_ is the histogram difference value which is the difference between (*i* + 1)th and (*i* + 2)th frames.

The output variable is

- transition with linguistic values no (NO), abrupt (AB), and gradual (GR).
- Variable transition is the type of transition that can occur from one frame to another. no represents the frame where there is no transition.

The rule base consists of 28 rules of the form as in [6]. In Table 1, rules for detecting no transition (frame without any transition) are given. For detecting gradual transition and abrupt transitions, the rules are provided in Tables 2 and 3, respectively.

### 4.2. Optimization with Genetic Algorithm

GA will be used to find the range of values of the membership function. We use the triangular membership function. The values of the input variables HD_*i*_, HD_*i*−1_, and HD_*i*+1_ range from 0 to 10. The values of the output variable are 0, 5, and 10 for no transition, gradual transition, and abrupt transition, respectively.

#### 4.2.1. Initialization

The unknown variables in this problem are the lengths of the bases of the five membership functions negligible, small, significant, large, and huge which will be same for the three input variables HD_*i*_, HD_*i*−1_, and HD_*i*+1_.

We will use 6-bit binary string to define the base of each five membership functions. The five strings, each of 6 bits, are then concatenated to form a 30-bit string which will be a solution for the population.

#### 4.2.2. Evaluation

The strings are mapped/encoded to values representing the lengths of the bases of the membership functions. This mapping process is computed using the following equation:

(2) $$\begin{array}{c} base^{\left(i\right)}=C_{min}^{\left(i\right)}+\frac{d}{2^{L}-1}\left(\;C_{max}^{\left(i\right)}-C_{min}^{\left(i\right)}\;\right), \end{array}$$

where *C*_min_^(*i*)^ and *C*_max_^(*i*)^ are user-defined constants and they are usually chosen as the minimum and the maximum value of the variable. *d* is the decimal value of each substring, *L* is the number of bits in each substring, and base^(*i*)^ is the *i*th base of the membership functions.

In the beginning, the GA randomly creates a population of 10 strings. For a string, the five bases of the five membership functions are calculated using (2).

Using the bases, we then find the initial, middle, and the final value (i.e., *a*, middle, and *b*) of the triangular membership functions of the linguistic values as given in Table 4.

*a*, middle, and *b* are the initial, middle, and the final value of the triangular membership functions of the linguistic values. *xd* is the fuzziness index which is a constant.

We then find the degree of the membership of the values in Table 6 using the rules. Using the degree of membership of the values in a rule, we then find the weight of the rule.

We have the following rule:

if(HD_*i*_ is huge) and (HD_*i*−1_ is negligible) and (HD_*i*+1_ is negligible) then *B*(*i*) is abrupt.

We find the degree of membership of the values contained in the rule as follows:

 deg of mem for HD_*i*_ = huge(input1, *a*_huge_, *b*_huge_, middle_huge_);
 deg of mem for HD_*i*−1_ = negligible(input2, *a*_neg_, *b*_neg_, middle_neg_);
 deg of mem for HD_*i*+1_ = negligible(input3, *a*_neg_, *b*_neg_, middle_neg_).

We then find the weight of rule *i* as follows:

(3) weighti=deg  of  mem  for  HDi∗deg  of  mem  for  HDi−1∗deg  of  mem  for  HDi+1.

In this way, we then find the weight of all the 28 rules. Using the weights, we then compute the crisp output for row *i* input values in Table 6 for a string/solution:

(4) crisp  outputi=weight1∗v1+weight2∗v2+⋯+weight28∗v28weight1+weight2+weight3+⋯+weight28,

where *v*_1_, *v*_2_, *v*_3_,…, *v*_28_ are preset values determined by us which is either 0, 5, or 10.

The sum of the squares of the above difference between crisp  output_*i*_ and actual  output_*i*_ for all the values in Table 6 becomes the fitness equation. The equation is shown as follows:

(5) $$\begin{array}{c} fitness=\overset{n}{\underset{i=1}{\sum }}{\left(\;crisp\;\;output_{i}-actual\;\;output_{i}\;\right)}^{2}. \end{array}$$

The fitness is subtracted from 1000 to convert the function from minimization to a maximization problem.

The above processes are repeated for all the strings/solutions of the population to find the fitness of all the strings.

#### 4.2.3. Selection

We then choose a set of strings whose fitness value is greater than some specific number.

#### 4.2.4. Reproduction

The population is modified using operators, namely, crossover and mutation.

These whole processes (evaluation, selection, and reproduction) are repeated for many generations and finally we then choose the bit string with largest fitness value.

This string with the largest fitness value will give the most optimal range of values for all the membership functions of the linguistic values.

After the GA finds the optimal values for the membership functions of the Fuzzy Logic system, the rule evaluation and the defuzzification procedure of the fuzzy system will start.

### 4.3. Rule Evaluation

**Figure 1:**
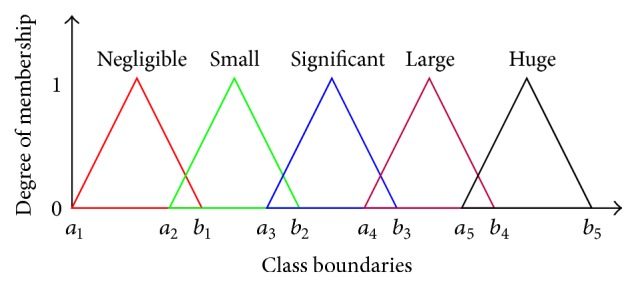
Fuzzy categories.

We need to find the degree of membership of the linguistic values of the input variables of the fuzzy system in the range of 0 to 1. We used the triangular membership function to find the degree of membership for the input variables. As shown in Figure 1, *a*_1_ to *a*_5_ and *b*_1_ to *b*_5_ are the range of values for a variable of a particular linguistic value.

### 4.4. Defuzzification

To find the crisp or actual output which is either no transition, gradual, or abrupt, we calculate the weights of the set of rules of the fuzzy system using the degree of membership.

Finally, we can calculate the crisp output by using (4).

## 5. Experimental Results and Discussion

### 5.1. Dataset

TRECVID 2001 video dataset for shot boundary detection is used for experimental results. TRECVID provides a set of video test data in MPEG compressed for video segmentation. TRECVID 2001 test video data is available on the* Open Video Project*. The details of the videos are given in Table 5.

### 5.2. Discussion

For discussion of the proposed system, two videos from the TRECVID 2001, namely,* Airline Safety (D5)* and* Perseus Global Watcher (D6)*, are used.  Table 7 shows the strings of the first generation GA operation with their decimal values, base values, value range of the membership function, and the fitness value. The strings are sorted according to their fitness value. The fitness is calculated as a difference between the actual outputs of some input data as shown in Table 6 and the crisp output of the same input data calculated using the membership function optimized by GA.  Table 8 shows the string with largest fitness value in different generations. We can see from the table, as the generation increases, that the fitness also increases.

**Figure 2:**
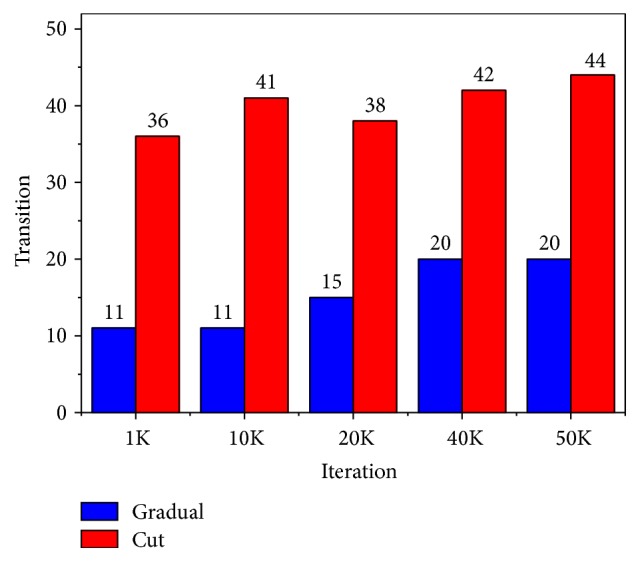
It shows result for shot boundary detection for the video “*Airline Safety and Economy*” for different iterations.

**Figure 3:**
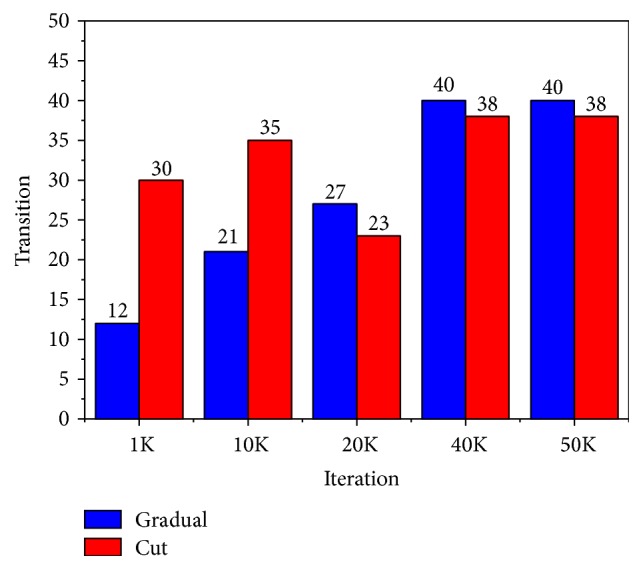
It shows result for shot boundary detection for the video “*Perseus Global Watcher*” for different iterations.

Figures 2 and 3 show the graph of shot boundary detection of two videos by our Fuzzy-GA system. The *x*-axis represents the iteration/generation of the GA operations. The *y*-axis represents the gradual and abrupt transitions of the video frames by our Fuzzy-GA application. We can see from the graph, as the iteration/generation increases, that the detection of the transition of the frames also increases.

In Figure 2, it is observed that, using the range of the membership function value obtained in 50000 (5K) iteration/generation of the GA optimization given in Table 8, our proposed system detects 20 gradual transitions and 44 abrupt transitions. The actual gradual and abrupt transitions of the video are 26 and 45, respectively, as given in Table 5.

In Figure 3, 40000 (40K) iterations/generations of our proposed system can detect 40 gradual transitions and 38 abrupt transitions which are out of actual 45 gradual transitions and 40 abrupt transitions as given in Table 5.

**Figure 4:**
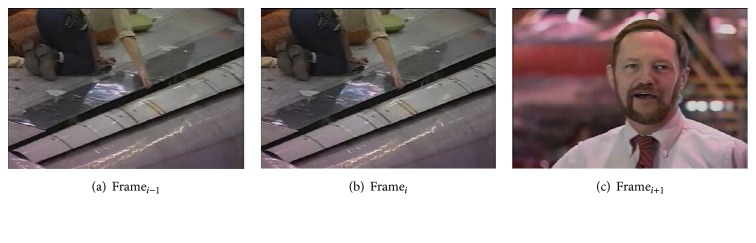
Showing abrupt transition.

Figures 4(a), 4(b), and 4(c) show three frames with abrupt transition of a video. The frame numbers of Figures 4(a), 4(b), and 4(c) are 6359, 6360, and 6361, respectively. The values of the input variables of the fuzzy system of this abrupt transition of the frames are as follows:

(1) HD_*i*_ = 3.127, (2) HD_*i*−1_ = 0.3743, and (3) HD_*i*+1_ = 0.7139.

Using the membership function value range of 10000 generation shown in Table 8, we then find the degree of membership of the linguistic values of the input variables present in the rules. We then calculate the weights of the set of rules using the degrees of membership. The weights of the 28 rules starting from rule number 0 are 0, 0, 0, 0, 0.0355, 0.0079, 0, 0, 0, 0, 0, 0, 0, 0, 0, 0, 0, 0, 0, 0, 0, 0, 0, 0, 0, 0, 0, 0, respectively. Finally, using the weights, we calculate the crisp output. crisp output = 0.4340/0.0434 = 10.00, which indicates abrupt transition.

**Figure 5:**
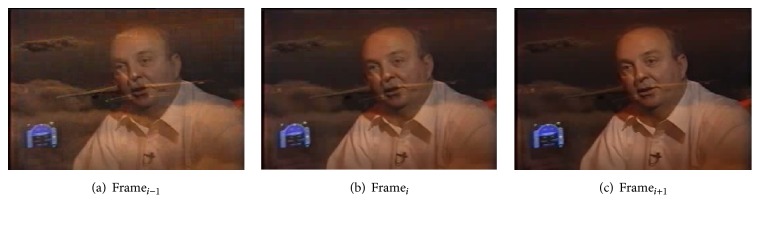
Showing gradual transition.

Figures 5(a), 5(b), and 5(c) show frames with gradual transition of the video (in case of dissolve). The frame numbers of 8, 9, and 10 are 4675, 4676, and 4677, respectively. The values of the input variables of the fuzzy system of this abrupt transition of the frames are as follows:

(1) HD_*i*_ = 3.088, (2) HD_*i*−1_ = 2.494, and (3) HD_*i*+1_ = 3.549.

Using the membership function value range of 10000 generations shown in Table 8, we then find the degree of membership of the linguistic values of the input variables present in the rules. We then calculate the weights of the set of rules using the degrees of membership. The weights of the 28 rules starting from rule number 0 are 0, 0, 0, 0, 0, 0, 0, 0, 0, 0, 0, 0, 0, 0, 0.2089, 0.0514, 0, 0, 0.1107, 0, 0, 0, 0, 0, 0, 0, 0, 0. Finally, using the weights, we calculate the crisp output. crisp output = 1.8545/0.3709 = 5.00, which indicates gradual transition.

**Figure 6:**
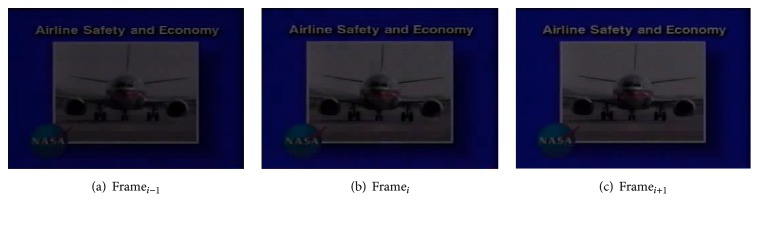
Showing gradual transition.

Similarly, Figures 6(a), 6(b), and 6(c) show another gradual transition (i.e., fade transition) which occurs between frames 4, 5, and 6, respectively. The values of the input variables of the fuzzy system of this abrupt transition of the frames are as follows:

(1) HD_*i*_ = 3.204, (2) HD_*i*−1_ = 2.636, and (3) HD_*i*+1_ = 2.409.

Using the membership function value range of 10000 generations shown in Table 8, we then find the degree of membership of the linguistic values of the input variables present in the rules. We then calculate the weights of the set of rules using the degrees of membership. The weights of the 28 rules starting from rule number 0 are 0, 0, 0, 0, 0, 0, 0, 0, 0, 0, 0, 0, 0, 0, 0.2939, 0.0829, 0, 0.2464, 0.0639, 0, 0, 0, 0, 0, 0, 0, 0, 0. Finally, using the weights, we calculate the crisp output. crisp output = 3.4359/0.6872 = 5.00, which indicates a gradual transition.

**Figure 7:**
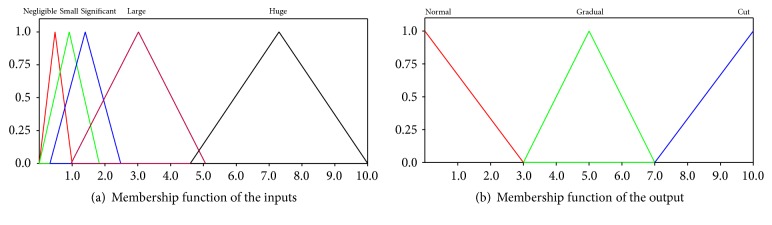
Fuzzy membership functions for input and output.

A pictorial representation of the fuzzy membership functions for inputs and output using the bases of 40K iterations or generations of the Genetic Algorithm is shown in Figure 7.

### 5.3. Evaluation

Recall, precision, and *F*1score parameters are used for evaluation of the proposed system which is given in

(6) Recall=NCNC+NM,Precision=NCNC+NF,F1score=2∗Recall∗PrecisionRecall+Precision.

The proposed system is compared with the latest techniques SBD using SVD and pattern matching [10] and SBD using Color Feature [9] and shows better performance in terms of *F*1score parameter. A comparison of the computational time is also provided in Table 9.

The computational time of the proposed system for all the videos in Table 5 is provided in Table 10. For each iteration/generation, the computational time includes the approximate time taken in seconds by the GA process, feature extraction, and the shot detection of the proposed system for all the videos.

In Table 11, recall, precision and *F*1score are represented by *R*, *P*, and *F*1, respectively.

## 6. Conclusion

This paper proposed a shot boundary detection using Genetic Algorithm and Fuzzy Logic. In this proposed system, GA is used as an optimizer for the fuzzy system. The GA system uses a preobserved actual input output values of shot boundaries for some videos for calculating the range of fuzzy membership values for the fuzzy system. The fuzzy system is used as a classifier which classifies the frames into abrupt and gradual transitions by using GA as optimizer. Normalized Color Histogram Difference is used for feature extraction and for finding the differences between two consecutive frames in a video. From the experimental result, it is observed that the detection of shot boundaries increases with increase in iteration or generation of the GA optimization process. Experimental results show that the proposed system yields better results and low computational time as compared with the latest techniques.

## Figures and Tables

**Table 1:** Rules for detecting no transition.

Sl. number	Rules for detecting no transition
1	if(HD_*i*_ is negligible) then *B*(*i*) is no
2	if(HD_*i*_ is small) then *B*(*i*) is no

**Table 2:** Rules for detecting gradual transition.

Sl. number	Rules for detecting gradual transition
1	if(HD_*i*_ is significant) and (HD_*i*−1_ is significant) then *B*(*i*) is gradual

2	if(HD_*i*_ is significant) and (HD_*i*−1_ is large) then *B*(*i*) is gradual

3	if(HD_*i*_ is significant) and (HD_*i*−1_ is huge) then *B*(*i*) is gradual

4	if(HD_*i*_ is significant) and (HD_*i*+1_ is significant) then *B*(*i*) is gradual

5	if(HD_*i*_ is significant) and (HD_*i*+1_ is large) then *B*(*i*) is gradual

6	if(HD_*i*_ is significant) and (HD_*i*+1_ is huge) then *B*(*i*) is gradual

7	if(HD_*i*_ is large) and (HD_*i*−1_ is significant) then *B*(*i*) is gradual

8	if(HD_*i*_ is large) and (HD_*i*−1_ is large) then *B*(*i*) is gradual

9	if(HD_*i*_ is large) and (HD_*i*−1_ is huge) then *B*(*i*) is gradual

10	if(HD_*i*_ is large) and (HD_*i*+1_ is significant) then *B*(*i*) is gradual

11	if(HD_*i*_ is large) and (HD_*i*+1_ is large) then *B*(*i*) is gradual

12	if(HD_*i*_ is large) and (HD_*i*+1_ is huge) then *B*(*i*) is gradual

13	if(HD_*i*_ is huge) and (HD_*i*−1_ is significant) then *B*(*i*) is gradual

14	if(HD_*i*_ is huge) and (HD_*i*−1_ is large) then *B*(*i*) is gradual

15	if(HD_*i*_ is huge) and (HD_*i*−1_ is huge) then *B*(*i*) is gradual

16	if(HD_*i*_ is huge) and (HD_*i*+1_ is significant) then *B*(*i*) is gradual

17	if(HD_*i*_ is huge) and (HD_*i*+1_ is large) then *B*(*i*) is gradual

18	if(HD_*i*_ is huge) and (HD_*i*+1_ is huge) then *B*(*i*) is gradual

**Table 3:** Rules for detecting abrupt transition.

Sl. number	Rules for detecting abrupt transition
1	if(HD_*i*_ is huge) and (HD_*i*−1_ is negligible) and (HD_*i*+1_ is negligible) then *B*(*i*) is abrupt

2	if(HD_*i*_ is huge) and (HD_*i*−1_ is negligible) and (HD_*i*+1_ is small) then *B*(*i*) is abrupt

3	if(HD_*i*_ is huge) and (HD_*i*−1_ is small) and (HD_*i*+1_ is negligible) then *B*(*i*) is abrupt

4	if(HD_*i*_ is huge) and (HD_*i*−1_ is small) and (HD_*i*+1_ is small) then *B*(*i*) is abrupt

5	if(HD_*i*_ is large) and (HD_*i*−1_ is negligible) and (HD_*i*+1_ is negligible) then *B*(*i*) is abrupt

6	if(HD_*i*_ is large) and (HD_*i*−1_ is negligible) and (HD_*i*+1_ is small) then *B*(*i*) is abrupt

7	if(HD_*i*_ is large) and (HD_*i*−1_ is small) and (HD_*i*+1_ is negligible) then *B*(*i*) is abrupt

8	if(HD_*i*_ is large) and (HD_*i*−1_ is small) and (HD_*i*+1_ is small) then *B*(*i*) is abrupt

**Table 4:** Membership function calculation for GA.

Membership	Range
Negligible	*a*_neg_ = 0
	$middle_{neg}=\frac{a_{neg}+b_{neg}}{2}$
	*b*_neg_ = base(neg)

Small	*a*_small_ = *b*_neg_ − *xd*
	$middle_{small}=\frac{a_{small}+b_{small}}{2}$
	*b*_small_ = *a*_small_ + base(small)

Significant	*a*_sig_ = *b*_small_ − *xd*
	$middle_{sig}=\frac{a_{sig}+b_{sig}}{2}$
	*b*_sig_ = *a*_sig_ + base(sig)

Large	*a*_large_ = *b*_sig_ − *xd*
	$middle_{large}=\frac{a_{large}+b_{large}}{2}$
	*b*_large_ = *a*_large_ + base(large)

Huge	*a*_huge_ = 10 − base
	$middle_{huge}=\frac{a_{huge}+b_{huge}}{2}$
	*b*_huge_ = *a*_huge_ + base(huge)

**Table 5:** Description of TRECVID 2001 test data.

Videos	Frames	Transitions			Sources
		Abrupt	Gradual	Total	
*D*2	16586	42	31	73	* NASA 25th Anniversary *
*D*3	12304	39	64	103	
*D*4	31389	98	55	153	

*D*5	12508	45	26	71	* Airline Safety *

*D*6	13648	40	45	85	* Perseus Global Watcher *

**Table 6:** Observed actual input output data.

Sl. number	HD_*i*_	HD_*i*−1_	HD_*i*+1_	Output
1	8.647	0.3216	1.107	10
2	7.746	1.716	0.8082	10
3	6.751	0.646	1.445	10
4	1.845	0.2521	0.7028	10
5	2.536	0.2865	0.7282	10
6	4.57	0.5302	0.939	10
7	5.54	0.2618	1.19	10
8	3.974	0.1552	1.333	10
9	0.5401	—	—	0
10	0.3632	—	—	0
11	0.6088	—	—	0
12	0.7728	—	—	0
13	2.49	—	4.654	5
14	1.537	—	1.859	5
15	2.926	2.14	—	5
16	3.293	1.39	—	5
17	3.305	—	2.089	5
18	1.741	—	1.026	5
19	4.654	2.49	—	5
20	7.048	—	6.441	5
21	3.621	—	2.462	5
22	4.522	1.928	—	5

**Table 7:** First generation data of GA.

Sl. number	String	Decimal value					Base value					Membership function value range					Fitness value
		*d*1	*d*2	*d*3	*d*4	*d*5	*b*1	*b*2	*b*3	*b*4	*b*5	Negligible	Small	Significant	Large	Huge	
1	011110 010101 011010 000001 010010	30	21	26	1	18	3.428571	3.000000	3.238095	2.047619	2.857143	0.000000 to 3.428571	2.428571 to 5.428571	3.928571 to 7.166667	5.666667 to 7.714286	7.142857 to 10.000000	218.243694

2	011000 101001 001000 010100 010001	24	41	8	20	17	3.142857	3.952381	2.380952	2.952381	2.809524	0.000000 to 3.142857	2.142857 to 6.095238	4.595238 to 6.976190	5.476190 to 8.428571	7.190476 to 10.000000	189.100768

3	000100 010110 101011 111101 100101	4	22	43	61	37	2.190476	3.047619	4.047619	4.904762	3.761905	0.000000 to 2.190476	1.190476 to 4.238095	2.738095 to 6.785714	5.285714 to 10.190476	6.238095 to 10.000000	182.306569

4	111011 001000 101101 110010 110001	59	8	45	50	49	4.809524	2.380952	4.142857	4.380952	4.333333	0.000000 to 4.809524	3.809524 to 6.190476	4.690476 to 8.833333	7.333333 to 11.714286	5.666667 to 10.000000	174.342243

5	110011 101010 100000 011000 110111	51	42	32	24	55	4.428571	4.000000	3.523810	3.142857	4.619048	0.000000 to 4.428571	3.428571 to 7.428571	5.928571 to 9.452381	7.952381 to 11.095238	5.380952 to 10.000000	144.445258

6	101110 101101 001011 001001 101011	46	45	11	9	43	4.190476	4.142857	2.523810	2.428571	4.047619	0.000000 to 4.190476	3.190476 to 7.333333	5.833333 to 8.357143	6.857143 to 9.285714	5.952381 to 10.000000	144.180334

7	110010 001011 100100 100011 000111	50	11	36	35	7	4.380952	2.523810	3.714286	3.666667	2.333333	0.000000 to 4.380952	3.380952 to 5.904762	4.404762 to 8.119048	6.619048 to 10.285714	7.666667 to 10.000000	142.464198

8	101110 100010 110110 111011 001100	46	34	54	59	12	4.190476	3.619048	4.571429	4.809524	2.571429	0.000000 to 4.190476	3.190476 to 6.809524	5.309524 to 9.880952	8.380952 to 13.190476	7.428571 to 10.000000	123.074104

9	111001 111010 101011 010010 111100	57	58	43	18	60	4.714286	4.761905	4.047619	2.857143	4.857143	0.000000 to 4.714286	3.714286 to 8.476190	6.976190 to 11.023810	9.523810 to 12.380952	5.142857 to 10.000000	59.725061

10	110110 111001 000100 011101 000111	54	57	4	29	7	4.571429	4.714286	2.190476	3.380952	2.333333	0.000000 to 4.571429	3.571429 to 8.285714	6.785714 to 8.976190	7.476190 to 10.857143	7.666667 to 10.000000	51.660022

**Table 8:** String with highest fitness of five generations.

Sl. Number	String	Generation	Membership function value range					Fitness value
			Negligible	Small	Significant	Large	Huge	
1	000010	1000	0.000000 to 2.095238	1.095238 to 3.142857	1.642857 to 3.642857	2.142857 to 7.095238	7.238095 to 10.000000	252.181591
	000001							
	000000							
	111110							
	010000							

2	000000	10000	0.000000 to 1.500000	0.500000 to 2.507937	1.007937 to 3.079365	1.579365 to 6.761905	5.515873 to 10.000000	263.203823
	001000							
	001001							
	111010							
	101111							

3	000010	20000	0.000000 to 1.126984	0.126984 to 1.444444	0.055556 to 4.690476	3.190476 to 7.873016	8.619048 to 10.000000	282.889190
	000101							
	111011							
	111010							
	000110							

4	000000	40000	0.000000 to 1.000000	0.000000 to 1.825397	0.325397 to 2.468254	0.968254 to 5.015873	4.619048 to 10.000000	333.609281
	001101							
	010010							
	110000							
	000110							

5	000000	50000	0.000000 to 1.000000	0.000000 to 1.825397	0.325397 to 3.039683	1.539683 to 5.587302	5.019048 to 10.000000	364.744095
	001101							
	011011							
	110000							
	000110							

**Table 9:** Comparison of the SBD using color feature [9] with the proposed system.

Videos	SBD using color feature [9]				Proposed system			
	Time (sec)	Recall	Precision	*F*1score	Time (sec)	Recall	Precision	*F*1score
*D*2	1310	0.928	0.951	0.939	30	0.952	0.889	0.919
*D*3	900	0.821	0.864	0.842	21	0.846	0.805	0.825
*D*4	2467	0.826	0.900	0.861	318	0.878	0.935	0.906
*D*5	1160	0.844	0.844	0.844	22	0.978	0.917	0.946
*D*6	2042	0.925	0.973	0.948	24	1.000	0.889	0.941
Average	1575	0.868	0.906	0.886	83	0.931	0.887	0.907

**Table 10:** Computation time of the proposed system.

Methods	Computation time (in secs approximately)
Proposed method with 1K iteration	491
Proposed method with 10K iteration	895
Proposed method with 20K iteration	1598
Proposed method with 40K iteration	2388
Proposed method with 50K iteration	3023
Average	1679

**Table 11:** Comparison of the SBD using SVD and pattern matching [10] with the proposed system.

Videos	SBD using SVD and pattern matching [10]						Proposed system					
	Abrupt			Gradual			Abrupt			Gradual		
	*R*	*P*	*F*1	*R*	*P*	*F*1	*R*	*P*	*F*1	*R*	*P*	*F*1
*D*2	0.905	0.905	0.905	0.935	0.725	0.817	0.952	0.889	0.919	0.806	0.833	0.819
*D*3	0.667	0.867	0.754	0.734	0.940	0.824	0.846	0.805	0.825	0.764	0.942	0.844
*D*4	0.888	0.897	0.892	0.727	0.741	0.734	0.878	0.935	0.906	0.727	0.816	0.769
*D*6	0.950	0.974	0.962	0.844	0.927	0.884	1.000	0.889	0.941	0.844	0.864	0.854
Average	0.853	0.912	0.877	0.810	0.833	0.814	0.919	0.880	0.898	0.785	0.864	0.822

## References

[1] Koprinska I., Carrato S. (2001). Temporal video segmentation: a survey. *Signal Processing: Image Communication*.

[2] Thounaojam D. M., Trivedi A., Manglem Singh K., Roy S., Mohapatra D. P., Patnaik S., Mohapatra D. P. (2014). A survey on video segmentation. *Intelligent Computing, Networking, and Informatics: Proceedings of the International Conference on Advanced Computing, Networking, and Informatics, India, June 2013*.

[3] Smeaton A. F., Over P., Doherty A. R. (2010). Video shot boundary detection: seven years of TRECVid activity. *Computer Vision and Image Understanding*.

[4] Wang X., Wang S., Chen H. A fast algorithm for MPEG video segmentation based on macroblock.

[5] Little T. D. C., Ahanger G., Folz R. J. A digital on-demand video service supporting content-based queries.

[6] Jadon R. S., Chaudhury S., Biswas K. K. (2001). A fuzzy theoretic approach for video segmentation using syntactic features. *Pattern Recognition Letters*.

[7] Anjulan A., Canagarajah N. (2007). Object based video retrieval with local region tracking. *Signal Processing: Image Communication*.

[8] Baber J., Afzulpurkar N., Satoh S. (2013). A framework for video segmentation using global and local features. *International Journal of Pattern Recognition and Artificial Intelligence*.

[9] Zhang H., Hu R., Song L. A shot boundary detection method based on color feature.

[10] Lu Z.-M., Shi Y. (2013). Fast video shot boundary detection based on SVD and pattern matching. *IEEE Transactions on Image Processing*.

[11] Gong Y., Liu X. Video shot segmentation and classification.

[12] Sun X., Zhao L., Zhang M. A novel shot boundary detection method based on genetic algorithm-support vector machine.

[13] Chan C., Wong A. Shot boundary detection using genetic algorithm optimization.

[14] Fang H., Jiang J., Feng Y. (2006). A fuzzy logic approach for detection of video shot boundaries. *Pattern Recognition*.

[15] Gao X.-B., Han B., Ji H.-B., Kamel M., Campilho A. (2005). A shot boundary detection method for news video based on rough sets and fuzzy clustering. *Image Analysis and Recognition*.

[16] Küçüktunç O., Güdükbay U., Ulusoy Ö. (2010). Fuzzy color histogram-based video segmentation. *Computer Vision and Image Understanding*.

[17] Tolias Y. A., Panas S. M. (1998). Image segmentation by a fuzzy clustering algorithm using adaptive spatially constrained membership functions. *IEEE Transactions on Systems, Man, and Cybernetics—Part A: Systems and Humans*.

[18] Abdulghafour M. (2003). Image segmentation using fuzzy logic and genetic algorithms. *Journal of WSCG*.

[19] Pal G., Rudrapaul D., Acharjee S., Ray R., Chakraborty S., Dey N., Satapathy S. C., Govardhan A., Raju K. S., Mandal J. K. (2015). Video shot boundary detection: a review. *Emerging ICT for Bridging the Future—Proceedings of the 49th Annual Convention of the Computer Society of India CSI Volume 2*.

[20] Goldberg D. E., Holland J. H. (1988). Genetic algorithms and machine learning. *Machine Learning*.

[21] Goldberg D. E. (1989). *Genetic Algorithms in Search, Optimization and Machine Learning*.

